# Housing Conditions Differentially Affect Physiological and Behavioural Stress Responses of Zebrafish, as well as the Response to Anxiolytics

**DOI:** 10.1371/journal.pone.0034992

**Published:** 2012-04-11

**Authors:** Matthew O. Parker, Mollie E. Millington, Fraser J. Combe, Caroline H. Brennan

**Affiliations:** School of Biological and Chemical Sciences, Queen Mary University of London, London, United Kingdom; Université de Bordeaux and Centre National de la Recherche Scientifique, France

## Abstract

Zebrafish are a widely utilised animal model in developmental genetics, and owing to recent advances in our understanding of zebrafish behaviour, their utility as a comparative model in behavioural neuroscience is beginning to be realised. One widely reported behavioural measure is the novel tank-diving assay, which has been often cited as a test of anxiety and stress reactivity. Despite its wide utilisation, and various validations against anxiolytic drugs, reporting of pre-test housing has been sparse in the literature. As zebrafish are a shoaling species, we predicted that housing environment would affect their stress reactivity and, as such, their response in the tank-diving procedure. In our first experiment, we tested various aspects of housing (large groups, large groups with no contact, paired, visual contact only, olfactory contact only) and found that the tank diving response was mediated by visual contact with conspecifics. We also tested the basal cortisol levels of group and individually housed fish, and found that individually housed individuals have lower basal cortisol levels. In our second experiment we found ethanol appeared to have an anxiolytic effect with individually housed fish but not those that were group housed. In our final experiment, we examined the effects of changing the fishes' water prior to tank diving as an additional acclimation procedure. We found that this had no effect on individually housed fish, but appeared to affect the typical tank diving responses of the group housed individuals. In conclusion, we demonstrate that housing represents an important factor in obtaining reliable data from this methodology, and should be considered by researchers interested in comparative models of anxiety in zebrafish in order to refine their approach and to increase the power in their experiments.

## Introduction

Zebrafish (*Danio rerio*) are widely utilized as a developmental genetic model, and are fast becoming established as a model in behavioral neuroscience [1]–[4]. The utility of the species in this regard hinges on the implementation of behavioral assays with high construct validity and reliability [5]. High throughput in-vivo techniques that are possible in zebrafish are of immense value [6], but also are of particular relevance in terms of embracing the concept of the 3Rs (refinement, reduction and replacement of animals in research), in the sense that it allows us to minimise the exposure of animals to procedures thus refining the techniques [7], [8]. The tank diving assay potentially fulfills this, and is commonly used as a measure of anxiety response to novel environments [9]. Similar to an open field procedure in rodents, anxiety is operationally defined by how much time the animal spends in the bottom half [10], [11] or bottom third [12] of a novel tank, which is usually 70–85% of the first minute, and reducing thereafter (see [9] for a recent review). The point at which the fish ventures into the top portion of the tank has been inferred to be the point at which the fish feels safe enough to explore its new environment [9], [13]. In addition, a variety of other behavioural markers (freezing, erratic swimming patterns) also appear to be correlated with anxiety in the procedure (e.g., see [10], [11]); however, it is not clear what aspects of anxiety these behaviours may represent as, to date, there is no convincing dissociation of any of the features either genetically, pharmacologically or otherwise [14].

The anxiolytic effects of drugs have been demonstrated using the tank diving assay [12], [13]. Typical behaviour at successful doses have included less time spent at the bottom overall and a faster ascent to the top portion of the tank [12], [15]. This change in response to the novel tank supports the reliability of tank diving as an assay of anxiety in zebrafish.

Different protocols exist for tank diving (e.g., see [10]–[12], [16]) and there is consensus on a number of procedural points. First, it is essential that the fish is placed in a novel tank to carry out the tank dive [12]. In addition, it is important that the fish is acclimated to the room in which the dive will take place for at least one hour prior to the task commencing [11]. It is also essential that the water in which the fish are tank dived is taken from the same source as that in which the fish are housed, in order to ensure equilibration of temperature and salinity [11], [12]. However, despite these details being widely adopted, and despite the tank-diving procedure being widely implemented in zebrafish behavioural neuroscience, no one has yet systematically assessed the effects of housing conditions on the response to the task. Housing is known to affect stress levels, with overcrowding increasing serum cortisol levels [17], but despite this, studies sometimes lack detail in how animals were housed prior to experimental trials. This is a crucial consideration for two reasons: 1) zebrafish are a shoaling species, and as such, the response of an individually housed fish to the novel tank test (which is carried out in isolation from others) would be expected to be very different from that of a fish that had been removed from its shoal. 2) if this test is measuring stress reactivity or anxiety, rearing a fish in isolation would be expected to alter its stress reactivity, thus creating problems with test reliability.

There are numerous ways in which zebrafish can be housed in the aquarium, and for Experiment 1 we arranged different housing conditions, with varying levels of visual and olfactory contact available, and tested fish from all of the different groups in the novel tank diving test. There have been also been various attempts to validate the tank diving test as a measure of anxiety in zebrafish by exposing the animals to different anxiolytic drug preparations prior to the test being carried out. The results of these tests, while interesting from a construct validity standpoint, have often produced data that appear somewhat inconsistent. For example, [12] found uneven dose-response curves for anxiolytic drugs (buspirone, diazepam) on bottom duration in the task. In order to test this in the context of housing conditions, we tested our group housed and individually housed fish following brief immersion in 1% ethanol. Anxiolytic effects of ethanol were first described by Gerlai et al [18] as when fish dosed with an appropriate amount of ethanol are no longer reactive to novel environments or objects (also see [15]). In addition, recently a strong dose-dependent anxiolytic response to ethanol was found in the novel tank diving test, where zebrafish showed a robust reduction in bottom-dwelling following exposure to 1% ethanol [16]. In our third experiment, we tested the effects of altering pheromone levels in the water by carrying out a full water change prior to tank diving. Finally, we modelled the data from all the experiments (adding some additional datasets) to examine which aspects of the tank diving response changed during different housing conditions, in order to try to shed light on the mechanisms modulating the response.

## Results

### Experiment 1

**Figure 1:**
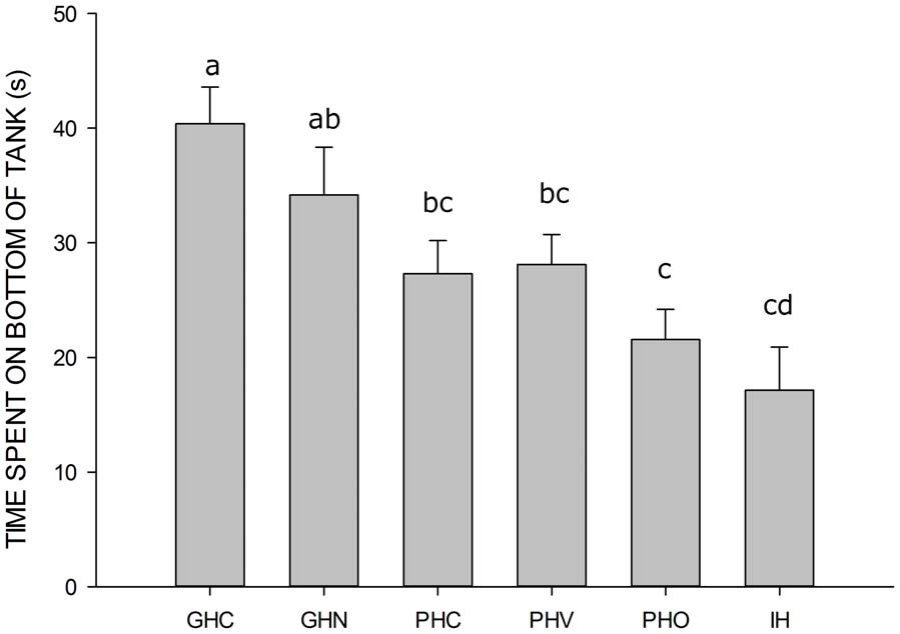
Time spent on the bottom of the novel tank according to housing conditions. Error bars represent SEM. Bars without shared letters differ significantly (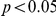).

**Figure 2:**
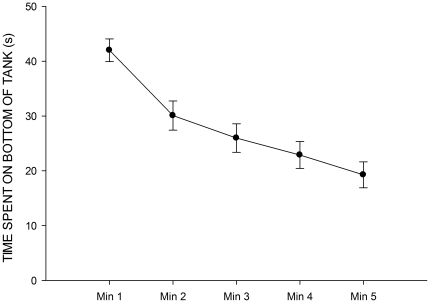
Time spent on the bottom of the novel tank across all housing conditions. Error bars represent SEM.

Fig. 1 displays the mean time spent on the bottom of the tank according to housing treatment. There was a reduction in time spent in the bottom third of the novel tank according to the level of grouping in the housing conditions, with the group housed fish showing the longest time on the bottom, and the individually housed fish spending the least time. This effect was confirmed with a linear mixed model (LMM) with time and group entered as fixed factors, ID nested in tank as random effects, and bottom duration as the response. There was a significant effect of group, 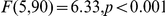 (see Fig. 1 for post-hoc analyses). There was also a significant effect of time, 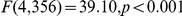, with time spent on the bottom of the tank decreasing as a function of time as expected (see Fig. 2), and this was consistent across housing conditions (housing
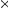
time interaction, 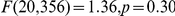).

**Figure 3:**
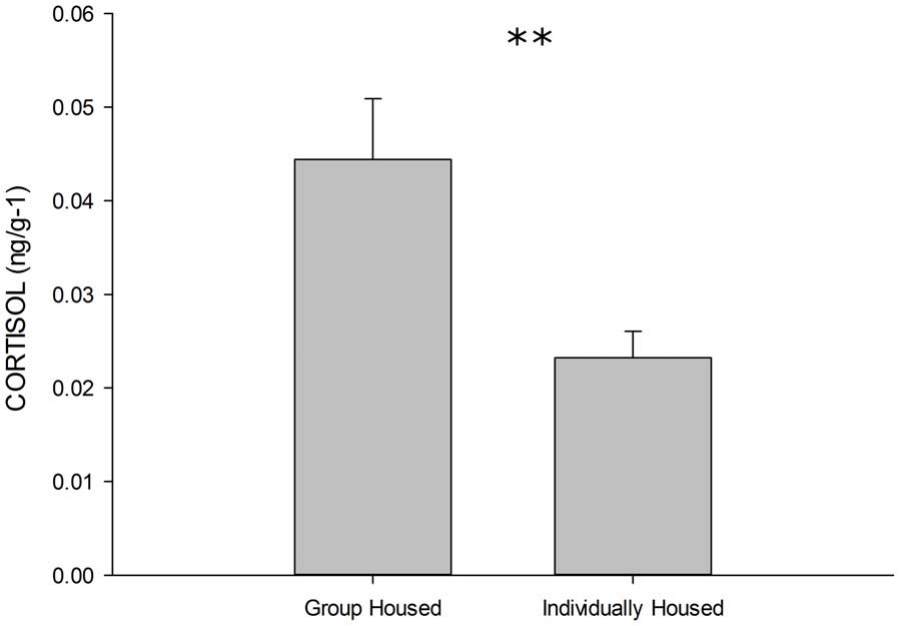
Baseline cortisol (ng/g^
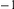
^) for individually and group housed fish. Error bars represent SEM. ** 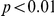.

Fig. 3 displays the baseline cortisol (ng/g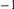) of fish according to their housing conditions. As is clear, group housed fish showed higher baseline cortisol than individually housed fish. An independent samples t-test confirmed that this difference was significant, 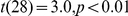.

### Experiment 2

**Figure 4:**
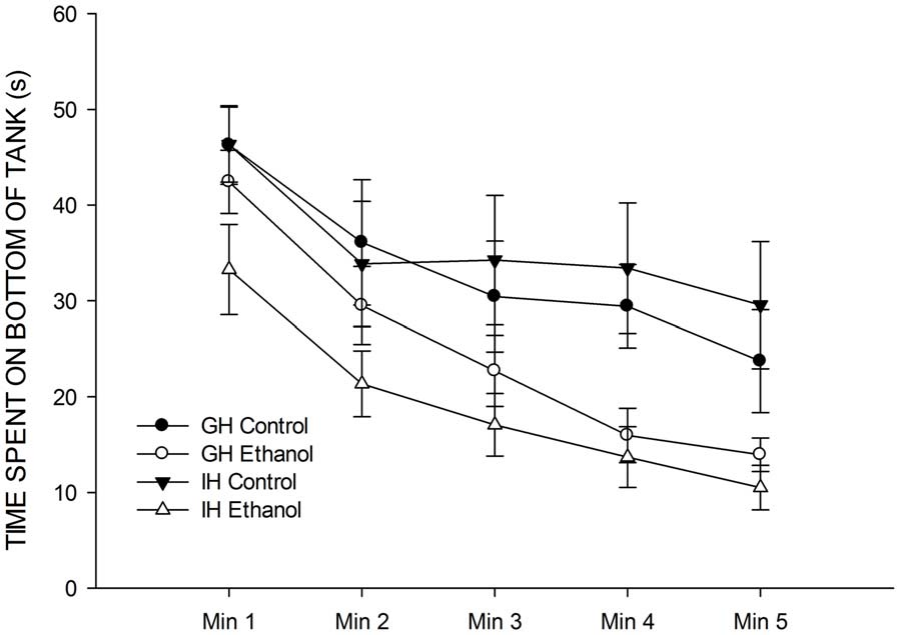
Time spent on the bottom of the novel tank for individually and group housed fish exposed to 1% ethanol or control, across five minute exposure to the novel tank. Error bars represent SEM.

Fig. 4 displays the time spent on the bottom of the tank during the tank dive for the group and individually housed fish exposed to 1% ethanol or fish H
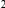
O for 20 minutes prior to the dive, or to fish water. It is clear that there was a treatment difference for the individually housed fish, but not for the group housed fish. This difference was confirmed with a LMM with time (5-levels), housing environment (individual vs group) and treatment (ethanol vs aquarium water) as fixed factors, ID nested in tank as a random effect (to account for between-tank effects) and time spent on the bottom of the tank as the response, which showed a significant housing
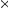
treatment interaction, 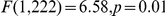. This was characterized by a significant difference between the 1% ethanol and saline treatments in the individually housed fish (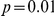), but no differences for the group housed fish (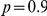). There was a significant main effect of time, 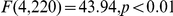, with time spent on the bottom of the tank decreasing in the expected fashion across the five minutes. There was also a significant effect of group, with individually housed fish again showing less time in the bottom of the tank, 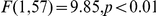). However, there were no interactions between treatment and time (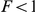) or group and time (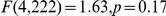), suggesting that time spent on the bottom of the tank decreased as expected, regardless of treatment/group.

### Experiment 3

**Figure 5:**
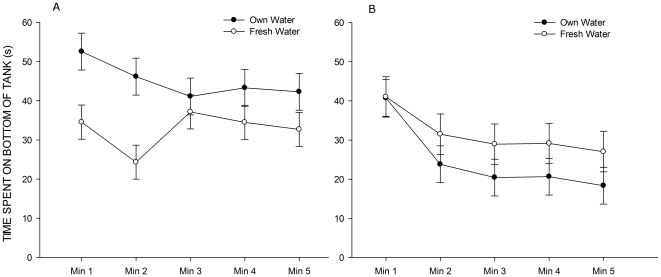
Time spent in the bottom of the novel tank for group (A) and individually (B) housed animals according to the water used during acclimation. Error bars represent SEM.

Fig. 5 illustrates a clear effect of moving the fish into fresh water prior to carrying out the tank diving response for the group housed fish, but not for the individually housed animals. This was confirmed with a LMM. There were main effects of time, 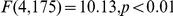 and group, 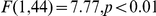. There was a significant group
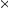
water interaction, 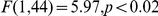, characterised by a significant change in tank diving response by the group housed fish when in fresh water (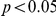) but not by the individually housed fish (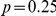). There were also time
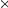
group and time
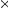
water interactions, 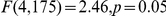 and 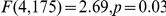, respectively, both characterised by changes in tank diving response of the group housed individuals when acclimated in fresh water.

### Data Modelling

**Figure 6:**
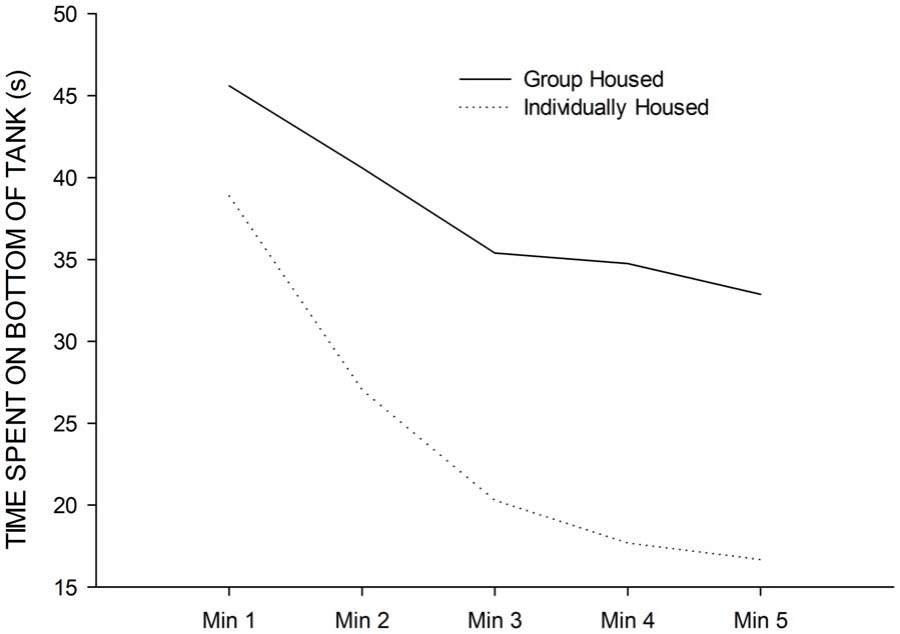
Estimated curves for group and individually housed fish. This graph represents the time spent bottom dwelling during the five minute exposure to the novel tank according to parameter estimates of a second order polynomial curve.

Three additional replicates of the different conditions (group and individually housed) were carried out in order to have sufficient data to model responses to the novel tank (see Fig. 6). We found that the change in bottom dwelling over the course of the five minute exposure to the novel tank decreased according to a second-order polynomial curve: 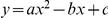. In this equation, the dependent variable, 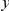, represents the time spent on the bottom of the tank, and the independent variable, 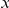, represents the time (i.e., minutes 1–5). Variables 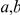, and 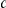 represent free parameters, which were estimated for each dataset, and compared between group and individually housed fish (see Table 1). As is clear, estimates of 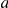 and 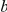 differ between the groups, but parameter 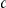 seems similar, and this was confirmed with Welch's two-sample t-tests (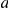: 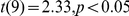; 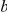: 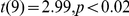; 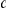: 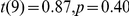). When we examined the tank diving curves for the other housing conditions from Experiment 1. The data are presented in Table 1. It seems that there is a trend for large group sizes (i.e., with the fish being tank dived after being removed from a larger group) showing lower parameter estimates for both 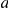 and 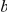 in the model, and there seems to be little difference amongst the pair housed fish, regardless of visual contact. This does suggest that the parameters 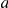 and 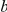 in the model may relate to aspects of group size, but with only one dataset representing each of the alternative housing conditions it would be unwise to over-interpret at this stage.

**Table 1:** Parameter estimates of tank diving model.

Housing	Dataset	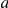	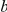	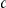
**Individual**	**1**	**1.56**	**16.44**	**57**
	2	1.31	13.2	44.3
	3	2.17	18.57	49.4
	4	2.39	20.89	63
	5	2.34	18.89	55.58
	6	1.25	10.56	49.37
	**MEAN**	**1.84**	**16.43**	**53.11**
	*SD*	*0.52*	*3.88*	*6.7*
**Group**	**1**	**0.97**	**10.9**	**55.5**
	2	1.14	10.24	53.65
	3	1.77	13.76	62.79
	4	0.65	8.25	48.78
	5	1.28	10.06	61.12
	*6	*-	*-	*-
	**MEAN**	**1.16**	**10.64**	**56.37**
	*SD*	*0.41*	*2*	*5.69*
**Pair: no visual contact**		1.13	11.31	43.7
**Pair: visual contact, no physical contact**		2.82	22.13	64.29
**Pair: contact**		1.49	15.24	57.35
**Group: no physical contact**		0.94	8.14	42.69

Parameters for the model were calculated from each dataset of individually and group housed fish. The table displays the parameter estimates, as well as the mean (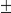
SD) for each group.

* Parameters for dataset 6 could not be fitted to the model. This dataset represented the tank diving response from the group housed fish which had their water changed prior to the tank dive.

## Discussion

The data from Experiment 1 demonstrated that housing affects the response of zebrafish on exposure to a novel tank. Specifically, we saw that despite the typically observed diving and slowly rising to the surface being seen regardless of housing, the latency to leave the bottom third of the tank appears to be very sensitive to housing conditions. We also found that group housed fish had higher resting cortisol levels than their individually housed counterparts. This is the first demonstration of either of these effects in the literature, and both have potentially important implications for researchers attempting to measure anxiety in zebrafish, in particular if they are using the novel tank diving task. There are several potential explanations for our findings. It may be that group housed fish have higher stress levels in general. Previously crowding has been shown to increase cortisol in fish [17]. This seems somewhat unlikely as our fish were housed in 5 L tanks in groups of 10, which would not normally be considered to be overcrowding. However, what seems more likely is that individually housing the fish caused a dampening of their stress reactivity. Chronic stress has been shown to cause downregulation of the HPA axis in other vertebrates [19], [20]. However, with teleost fish, the HPI axis (hypothalamic–pituitary–interrenal axis) is somewhat different from the mammalian HPA (hypothalamic–pituitary–adrenocortical axis) in that in the fish, this system is also utilised in osmoregulation [21]. As such, it is possible that the differences in housing reflected some subtle differences in water quality, rather than differential stress sensitivity. Nevertheless, the task poses very different challenges for group housed and individually housed fish, with the former responding to isolation, and the latter simply to a change in surroundings.

In Experiment 2, we repeated the extreme conditions (group housed vs. individually housed) in an attempt to characterise differences in the tank diving response to a mild anxiolytic, ethanol. We replicated the finding that individually housed fish showed the typical tank diving response, but spent less total time on the bottom of the tank as the group housed individuals. In addition, we found that the apparent efficacy of 1% ethanol as an anxiolytic in this context was dependent on housing, with the group housed fish showing no difference in their response to the novel tank, but the individually housed fish staying in the bottom third of the tank for less time. This finding was all the more intriguing in the light of the results of Experiment 1, as it seems to support the assumption that there may be different processes for the group and individually housed individuals which is driving the tank diving response. It may, for example, be that the individually housed fish are showing a mild anxiety response (i.e., as evidenced by the decrease in their bottom dwelling after exposure to an anxiolytic) and the group housed fish are reacting to changes in the environment, perhaps by engaging in search behaviour triggered by the change in water (i.e., from their group water to fresh). This may explain why this group did not react to the ethanol. In the final experiment, we tested this by changing the water prior to acclimation. We found that moving the fish into fresh water prior to carrying out the task abolished the tank diving response for the group housed fish, but not for the individually housed animals, suggesting that changing the water prior to tank diving virtually eliminates the typical tank diving response (i.e., gradual reduction in bottom dwelling) in the group housed fish, but has little effect on the individually housed animals.

In our first experiment, we found that individually housing fish results in the animals spending significantly less time bottom dwelling during the five minutes of the tank dive. This was true also for fish which were sharing water with a conspecific, but could not see them (our pair housed with olfactory contact group [PHO] group; see Fig. 1). To a certain extent, this was also true of fish housed in pairs (either with or without physical contact) which showed significant differences from both the group and the individually housed fish. It seems therefore that the response to the novel tank is mediated by the conditions in which the fish are kept. Others have shown that high stocking density can increase stress, i.e., as evidenced by high cortisol levels [17], but as far as we know, we are the first group to examine baseline cortisol as a result of individual housing in zebrafish. Somewhat surprisingly, we found that individually housed fish showed lower baseline cortisol than group housed. As discussed earlier, this may be the result of dampening of the HPI axis in the individually housed fish (as often reported with respect to the HPA axis in mammals [19], [20]), which may explain their response in the tank diving task being lower than that of the group housed fish. However, a mild anxiolytic dose of ethanol had no effect on the group housed, but did reduce tank diving in the individually housed, fish. Further, when the water was changed to fresh water prior to the tank dive being carried out, the idiosyncratic gradual rise to the surface was abolished in the group housed fish, but remained unaffected in the individually housed animals. As such, and alternative interpretation of these patterns could relate to zebrafish exhibiting different coping styles according to their dominance status within their social groups, and the fact that *passive* copers have higher post-stress cortisol levels than *active* copers [22]. It is therefore possible that the lower cortisol in the individually housed fish resulted from this group having altered HPI axes owing to the lack of social interactions. This would need to be investigated further before inference was made.

It seems likely that the anxiety caused by the tank diving procedure may not be the same in all cases. Rather, if it represents a measure of the fish sampling different aspects of the environment in terms of olfactory and visual cues, it would be expected that the extent of the response to the environment was dependent on the degree of change from the normal environment (i.e., that to which the fish was used). For example, the reason that the individually housed fish reliably showed a dampened response as compared to the group housed fish may be because the change of environment when introduced to the new tank was not as severe. In other words, the response shown by the individually housed fish represents a fairly mild stressor in the sense that it is sampling a new set of olfactory and visual cues, but for the group housed fish there are both of these factors, but also separation from their group. This is further evidenced by the results of Experiment 1 where we demonstrated that there was a gradual decrease in time spent bottom dwelling as a function of contact with conspecifics. Bencan et al [12] demonstrated that the width of the housing tank is a factor influencing bottom dwelling, with fish housed in narrower tanks (i.e., the same width as the tank diving tank) showing less bottom dwelling. Interestingly, their data showed that fish in the narrow-tank condition did not show the typical tank diving response, i.e., they appeared to spend an equal amount of time in the three sections of the tank across the 6 minute period. Although the authors interpreted this as being a muted response to the tank dive, owing to the familiarity of the novel tank, according to our model the tank diving response in their narrow-tank fish was abolished altogether, similar, in fact, to the effect we described from group housed fish placed in new water prior to the tank dive being carried out. When the data from all the experiments reported above were modelled (in combination with three additional datasets) it was apparent that there were differences in the parameter estimates that appeared to relate to group size prior to tank diving. The only case in which the data did not fit the model, however, was the condition in which the group-housed individuals were acclimated to the testing room in new water. This suggests that removal of the olfactory cues from their home tank altered the typical diving response, and merits further investigation.

In summary, we have demonstrated that the typically observed tank diving response, commonly used to assess anxiety and stress in zebrafish, is related specifically to the degree of change from the environment from which the fish has come. Further, we have demonstrated that the tank-diving test may be unreliable if housing conditions of the fish are not taken into account. This presents something of a conundrum, as it may compromise welfare to house the animals individually, but may produce more reliable results thus reducing the number of animals required. If group housing causes such robust and reliable differences in tank diving response, it is likely that the dynamics within particular groups are also important to the response of individuals within it. As such, if comparing between groups in the tank diving test (i.e., between mutant or wild-type strains) our data suggest that it is crucial that pseudoreplication is avoided in order to eliminate the possibility of erroneous results in this procedure. Zebrafish are becoming more widely utilised in behavioural neuroscience research, and part of their appeal rests in the 3Rs (refinement, reduction and replacement of animals in research). However, it may be that group housing, which would improve welfare and encourage naturalistic behaviours, may increase the required sample size in order to achieve sufficient statistical power to test hypotheses with an expectation of marginal or small effect sizes. In this sense, group housing may increase construct validity, but reduce reliability in cases where high levels of replication are necessary. In conclusion, we would urge researchers to consider group size and housing conditions in order to reduce animal use and refine the techniques, and based on our conclusions, we would suggest that fish are pair housed for at least two-weeks prior to tank diving in order to optimise the trade-off between construct validity, reliability and welfare.

## Materials and Methods

### Subjects

A total of 95 locally bred and reared short-fin wild type zebrafish from group housing tanks, initially on a recirculating AHAB system (Aquatic Ecosystems, Florida, USA), were used as subjects. Prior to the experiment starting, fish were organised into their housing conditions and left for 2 weeks. During this time, fish were fed three times each a day; twice with brine shrimp (morning and late afternoon) and a mid-day feed of flake food. All fish were adult (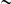
5 months old) at time of testing, and were separated into five different groups (see below for details). The fish were kept at 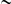
28
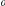
C on a 14 hr∶10 hr light∶dark cycle (lights on 9am) and housed in aquarium water (de-ionized water with added marine salts). All tanks were fitted with air-lines and regularly monitored for water quality. Tank water was changed weekly. Following completion of the experiment, all fish were returned to our breeding stock. A further 30 fish were used for the cortisol assay (n = 15 group housed, chosen at random from 6 tanks of n = 10 fish; n = 15 individually housed). This work was regulated by the United Kingdom Animals (Scientific Procedures) Act (1986), and was conducted with local ethical approval (Queen Mary University of London).

In experiment 1, we had the following allocation of fish: Group housed with contact (GHC; n = 14) were selected from three tanks (5 L), each containing 10 fish (i.e., n = 4–5 from each tank). Group housed with no contact (GHN; n = 10) were housed in two large tanks (100 L) and each fish was placed in a transparent divider (a plastic bottle with small perforations to allow water flow) such that it had no contact with other fish, but could see them. Pair housed with contact (PHC; n = 19) were housed together in a tank (1 L; height
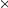
width
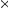
length: 10 cm
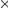
11 cm
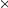
20 cm) with full contact. Pair housed with visual contact (PHV; n = 11) were housed together in a tank (1 L; height
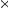
width
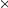
length: 10 cm
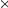
11 cm
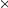
20 cm) with a transparent divider separating the two fish, such that they could see each other, but not have physical contact. Pair housed with olfactory contact only (PHO; n = 11) were pair housed in a tank (1 L; height
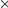
width
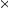
length: 10 cm
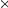
11 cm
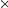
20 cm) with an opaque divider separating the two fish, such that olfactory cues could be detected, but no visual cues. Finally, individually housed fish (IH; n = 10) were housed individually in tanks (1 L; height
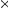
width
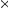
length: 10 cm
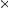
11 cm
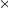
20 cm) with no visual or olfactory cues. All fish were kept in the housing conditions stated above for 2 weeks, prior to the tank diving tests being carried out.

The fish used in Experiment 2 were naive to the procedure. As before, they were housed in their experimental conditions for two weeks prior to the tank diving procedure being carried out. All husbandry procedures were the same as in Experiment 1. A total of 60 fish were used for this experiment (n = 29 group housed, selected from six groups of n = 10; n = 31 individually housed). Approximately half of the fish from each housing condition were assigned to the ethanol group (n = 15 group housed; n = 15 individually housed) and half to the control group (n = 14 group housed; n = 16 individually housed). The individually housed fish ere housed in 1 L tanks as in experiment 1 (height
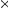
width
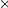
length: 10 cm
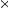
11 cm
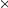
20 cm) and the group housed fish in larger 5 L tanks. After the experiment, all fish that had been given ethanol during the procedure were killed, and all those that had been given control treatment (i.e., no ethanol) were returned to our breeding stock.

The fish used in Experiment 3 were naive to the procedure. As before, they were housed in their experimental conditions for two weeks prior to the tank diving procedure being carried out. All husbandry procedures and housing procedures were the same as in experiments 1 and 2 for the individually and group housed fish. A total of 46 fish were used for this experiment (individually housed: n = 22; group housed: n = 24, selected from six groups of n = 10). Fish from each housing condition were assigned to the ‘fresh water’ group (n = 14 group housed; n = 10 individually housed) or the ‘own water’ group (n = 12 group housed; n = 12 individually housed). After the experiment, all fish were returned to our breeding stock.

### Experimental Design and Apparatus

**Figure 7:**
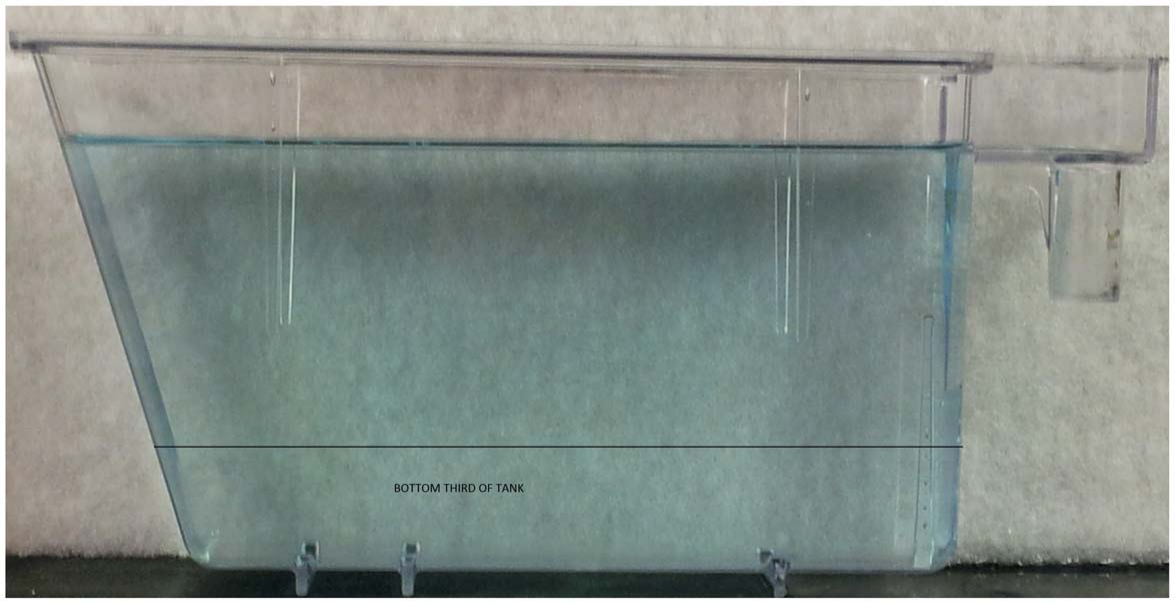
Tank used for novel tank-diving test. Fish were netted, placed in the tank and allowed to explore for five minutes. They were filmed during the exploration, and the amount of time spent in the top, middle and bottom of the tank was recorded, as well as the distance swum and velocity.

All experiments employed a a fully randomised (i.e., in terms of group allocation from original housing tanks in our aquarium) between-subjects design, with all fish taking part in the tank diving assay only once. The tank diving task was carried out in 1.5 L trapezoid tanks (15.2 height
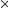
27.9 top
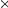
22.5 bottom
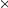
7.1 width cm) filled with aquarium treated water from the main aquarium supply (see Fig. 7). Care was taken to ensure that the water temperature of the novel tank was equilibrated to the home tank water temperature. For the drug treatment (Experiment 2), 1% ethanol was added to aquarium water (see Procedure section).

### Procedures

#### Experiment 1

Prior to tank diving, all fish were transported to our behavioural room in their housing tanks and acclimated to the conditions of the room for at least 1-hr. Following acclimation, fish were individually introduced to the tank diving apparatus, and filmed for five-min exploring the tank. Fish were tracked, and data were extracted, using EthoVision (Noldus, Netherlands). To ensure that the pre-testing experience of the fish was the same, we were careful to arrange the order of testing such that group-housed fish from the same tank were not tested consecutively, as the dipping of the net into the tank would potentially have caused stress for the other fish in the group.

For the cortisol analysis, fish were housed either in groups or individually (as above). Resting cortisol levels were assessed using a human salivary cortisol ELISA kit (Salimetrics) as previously described [11]. Fish were killed by immersion in ice, then frozen at −80
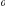
C until assay. Briefly, the fish were thawed and heads were removed. All samples were weighed and homogenized in 5 ml ice-cold PBS. 5 ml of diethyl ether was added and samples were centrifuged (7000 g) for 15 minutes, and the top (organic) layer was removed. This was repeated three times. The diethyl ether was evaporated overnight, and the resulting cortisol was reconstituted in 1 ml ice cold PBS. The ELISA was then performed in 96-well plates as per the manufacturer's instructions. Cortisol concentrations (ng/g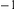) were determined from OD readings compared against manufacuter-provided standards. All samples were run in duplicate and the inter- and intra-assay coefficients of variation were 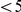
%.

#### Experiment 2

Prior to tank diving, fish were acclimated to the conditions of our behaviour room for 1 hour. In order to minimise handling, and to ensure that group housed fish were not removed from their group prior to tank diving, for the ethanol treatment we submerged 1 L clear plastic bottles, with either 300 ml of 1% ethanol solution or aquarium water in each group tank. By using the fishes' original tank water, we hoped to preserve any pheromones or other familiar odours the fish were accustomed to. The bottles also allowed visual contact with tank mates so as not to overly stress the fish prior to tank diving. Half of each housing group were placed in either the control (no ethanol exposure) or ethanol treated groups. Fish were placed in the ethanol or control bottles for 20 minutes. After that, fish were placed in a separate tank containing home tank water for two minutes to rinse off ethanol and then tested in the tank diving procedure immediately. For individually housed fish, they were exposed to ethanol added to their tank water, and were removed into a separate tank prior to tank diving in the same way as the group housed individuals. The order in which the fish were tested was fully counterbalanced according to housing conditions and ethanol treatment. The tank diving procedure was carried out as before, with data collected via EthoVision (Noldus, Netherlands).

#### Experiment 3

Fish from the ‘fresh water’ group were moved from their home tank into an identical new tank of fresh water immediately prior to acclimating them to the tank-diving room. The fish that remained in their home tank water were also netted and replaced in their own tank (i.e., with no water change) to control for potential effects of netting on their tank dive performance. Note that the water into which the fresh water fish were placed was taken from the aquarium water source, and as such was the same in terms of salinity and temperature as that from which the fish had been moved.

### Analysis

#### Statistical analysis

For Experiment 1, data were fitted to linear mixed models (LMM), with ‘housing conditions’ (six levels: GHC, GHN, PHC, PHV, PHO and IH) and time (five levels: mins 1–5) as fixed factors, and distance covered (cm) and velocity as covariates. Bottom duration (i.e., time spent in the bottom third of the tank) was entered as the response. Other authors (e.g., [10]) have often analysed a variety of response variables in the tank-diving test such as erratic swimming, freezing, etc., but here we chose to add these covariates to the main models in order to avoid multiple testing of these often highly correlated variables. In addition, it was clear that the behavioural response to stress in zebrafish can manifest as freezing or erratic swimming, and this seems to vary between subjects. In the absence of robust empirical fractionation of these behavioural responses, we feel it is unwise to use these measures to make between-group inference about the generalised stress response. We also entered ‘Fish’ nested in ‘Tank’ as a random effect in the model to avoid pseudo-replication with groups/paired fish. Cortisol data were generated from OD readings and normalised for weight. Group differences (individually housed vs group housed) were tested with a between-subjects t-test. Tank diving data were entered into a LMM with time (five levels), housing conditions (group vs individual) and ethanol treatment (1% ethanol vs control) as fixed factors and time spent on the bottom of the tank as the response. As before, distance travelled and velocity were added into the models as covariates, and fish ID nested in tank was added as a random effect to control for pseudo-replication. In Experiment 3, data were entered into a LMM with time (five levels), housing conditions (individual vs group) and water (fresh vs own) as fixed factors, ID nested in tank as a random effect (to account for between-tank effects) and time spent on the bottom of the tank as the response. Distance travelled and velocity were entered as covariates. Descriptive statistics are reported as mean 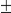 SEM unless otherwise indicated. Results of all statistical analyses are reported with respect to a type-1 error rate of 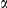 = 0.05 (post-hoc tests were conducted using Tukey's HSD). All statistical analyses were carried out in R version 2.12.2 (www.r-project.org).

#### Data modelling

Three additional datasets were collected following the completion of experiments 1–3 in order to have sufficient replicates for modelling. For these additional datasets, individually housed fish were kept for two weeks in 1.5 L tanks, and the group housed fish were kept in 5 L tanks, 10-fish to a tank prior to assay. All fish were then tank-dived as before. All data were then collated with the datasets from experiments 1–3. We plotted the tank diving responses (i.e., bottom duration over a 5 minute period) of individually and group housed fish independent of one another, and fitted regression lines. We then estimated the model parameters for each condition. We also compared the parameters from the model with those generated from the various manipulations in housing conditions during the experiments (see Experiment 1).
